# Surgical Stress Response: A Physiological Review of the Endocrine, Immune, and Metabolic Changes

**DOI:** 10.7759/cureus.100101

**Published:** 2025-12-26

**Authors:** Kirtish Acharya, Deepak Kumar Rout, Nilaykumar A Kapadia, Yash Satish Caroicar, Naresh Babu Karunakaran, Punit Patel, Harsiddh Thaker

**Affiliations:** 1 Department of Physiology, Fakir Mohan Medical College and Hospital, Balasore, IND; 2 Department of Psychiatry, Manipal Tata Medical College, Manipal Academy of Higher Education, Manipal, IND; 3 Department of Physiology, Banas Medical College and Research Institute, Palanpur, IND; 4 Department of Medicine, Goa Medical College and Hospital, Goa University, Bambolim, IND; 5 Department of Occupational Therapy, Sree Abirami College of Occupational Therapy, The Tamil Nadu Dr. M.G.R. Medical University, Coimbatore, IND; 6 Department of Community Medicine, Banas Medical College and Research Institute, Palanpur, IND; 7 Department of Physiology, Dr. N. D. Desai Faculty of Medical Science and Research, Dharmsinh Desai University, Nadiad, IND

**Keywords:** biomarkers, immunometabolism, neuroendocrine regulation, perioperative medicine, surgical stress response

## Abstract

Surgical intervention triggers a complex physiological cascade, also known as the surgical stress response, which entails well-coordinated immunological, metabolic, and neuroendocrine processes. This systematic review summarizes the existing evidence from 11 studies published between 2015 and 2025, investigating the underlying mechanisms, regulatory interactions, and clinical implications of this multifactorial response. Central stress pathway activation triggers immediate hormonal increase, stabilizing circulation and energy reserves, but chronic endocrine stimulation has the potential to increase metabolic and immune imbalances and results in a delayed recovery. The ensuing immune dynamics display an initial inflammatory phase followed by transient suppression, collectively influencing tissue repair and infection susceptibility. Parallel metabolic adaptations enhance energy production but often induce insulin resistance and protein catabolism, contributing to postoperative fatigue and functional decline. These interdependent mechanisms form a unified neuroendocrine-immune-metabolic network that can shift from adaptive to maladaptive under persistent stress. New evidence indicates that biomarkers such as cortisol, IL-6, glucose, and albumin serve as predictors of postoperative complications, including infection, impaired wound healing, and increased morbidity. Various perioperative interventions, such as the Enhanced Recovery After Surgery programs, immunonutrition, and metabolic administration, exhibit the possibility of maximizing such responses. The review combines both mechanistic and clinical perspectives to promote precision-based perioperative tactics to enhance surgical and recovery outcomes.

## Introduction and background

Although surgical intervention is a constituent component of the treatment of a wide spectrum of pathological states, it is one of the greatest physiological demands the human body must cope with [1]. Surgical tissue damage, hemorrhage, anesthesia, and tight interactions of hemodynamics are exceptionally orchestrated and dynamic stimuli that cause an exceptionally intricate cascade of neuroendocrine, immune, and metabolic responses, termed the surgical stress response [2]. This is an evolutionary adaptive mechanism of survival that is geared toward homeostasis and the mobilization of energy at the cost of tissue repair [3]. But when it is excessive or dysregulated, it may also lead to poor postoperative outcomes, including infection, poor wound healing, metabolic derangements, and even organ dysfunction [4].

Neuroendocrine activation is one of the initial responses. Nociceptive input caused by tissue trauma triggers the hypothalamic centers and leads to the activation of the hypothalamic-pituitary-adrenal (HPA) axis and the sympathetic-adrenal-medullary system [5]. This secretion of adrenergic hormones, cortisol, catecholamines, and antidiuretic hormone causes a cascade of physiological alterations [6]. Cortisol enhances gluconeogenesis and the immune system, and catecholamines enhance the heart output, blood supply, and skeletal muscle redistribution [7]. Initially, these endocrine adaptations are protective; however, when endogenous stress hormones continue to be secreted for a prolonged period, they may predispose patients to hyperglycemia, immunosuppression, and delayed recovery [8].

Meanwhile, it is accompanied by a profound immune system modulation. Rapid inflammatory response, evidenced by cytokine secretion, leukocyte activation, and endothelial signaling, is linked to surgical trauma [9]. Their function is essential for both tissue repair and pathogen protection; however, when dysregulated, they can trigger a systemic, maladaptive inflammatory response [10]. The ratio of pro- and anti-inflammatory mediators determines the surgical outcomes, and its absence leads to sepsis, poor wound healing, or extended hospitalization [11]. In addition, temporary disruption of adaptive immunity disrupts host defenses, leaving an individual vulnerable to nosocomial infections [12].

The metabolic aspect of its response emphasizes the systemic magnitude of the stress response. Increased metabolic demand following surgery due to a high rate of carbohydrate, lipid, and protein breakdown is what leads to the hypermetabolic condition [13,14]. Insulin resistance by actions of hormonal and cytokine signaling ensures the availability of glucose in crucial tissues without aggravating the hyperglycemia postoperatively, but hampering anabolic processes [15,16]. These metabolic alterations, along with alterations in substrate utilization and nitrogen imbalance, can contribute to muscle wasting, slower functional recovery, and convalescence unless addressed [17]. They also suggest the complexity of the interaction between endocrine and immune networks and the general impact on cellular physiology [18].

These are complex physiological changes that should be comprehended to maximize perioperative care [19]. Moderation of the intensity of surgical stress has been achieved by the use of technologies such as minimal invasive surgery, better recovery regimens, and patient-centered anesthetic plans, yet age, comorbidity, nutritional conditions, and genetic orientation determine patients' outcome conditioning [20]. It is possible to comprehend the fragile interconnection between the endocrine, immune, and metabolic reactions and translate it into particular interventions, including hormonal modulation, immunonutrition, and metabolic optimization, and have a positive effect on the patterns of postoperative recovery [21,22].

This is a field that has been studied for decades, but the knowledge remains fragmented. The majority of the literature has focused on analyzing separate parts of the stress response, including cortisol dynamics, cytokine patterns, or insulin resistance, without applying them to create a coherent physiological system. This is a compartmentalized way of looking at the interdependence of these responses and how they work together to affect recovery. Furthermore, newer discoveries in molecular biology and immunometabolism have also shown new mechanistic pathways, including neuroimmune cross-talk and metabolic-immune signaling, to be synthesized in the future. There is no established procedure for measuring the severity and clinical relevance of the surgical stress response, further complicating the translation into clinical practice. The gaps should be addressed by a systematic synthesis at the system level to support evidence-based perioperative approaches.

Objectives of the review

This systematic review summarizes existing knowledge on endocrine, immune, and metabolic responses to surgical stress, how they interact, and the clinical importance of these interactions. It further identifies the new therapeutic approaches and research priorities to be developed in the future to influence perioperative care and enhance the outcome of surgical procedures.

## Review

Reporting standards

This review followed the Preferred Reporting Items for Systematic Reviews and Meta-Analyses (PRISMA 2020) guidelines to ensure methodological rigor, transparency, and reproducibility [23]. The structure of the Methods section follows PRISMA recommendations, beginning with eligibility criteria and progressing through search strategy, study selection, data extraction, quality assessment, and risk of bias evaluation.

Eligibility criteria

Eligible studies included peer-reviewed research articles, systematic reviews, cross-sectional studies, cohort studies, case-control studies, randomized controlled trials (RCTs), and narrative or mechanistic reviews that examined endocrine, immune, or metabolic responses to surgical stress in human subjects. Studies were required to report perioperative biomarker fluctuations, hormonal responses, immune modulation, metabolic alterations, or associated clinical outcomes. Only English-language publications released between January 2015 and March 2025 were considered. Studies were excluded if they involved animal or in vitro models, were written in languages other than English, lacked full-text availability, or were editorials, commentaries, or opinion pieces without primary data or mechanistic synthesis. These criteria ensured that the included literature addressed human physiological responses relevant to surgical stress.

Search strategy

A comprehensive search was conducted across PubMed, Scopus, Web of Science, ScienceDirect, and Google Scholar. These databases were selected because of their extensive biomedical coverage and capacity to capture multidisciplinary research relevant to surgical stress physiology. The search spanned January 2015 to March 2025.

A combination of Medical Subject Headings and free-text keywords was used. An example PubMed search string was: “surgical stress response” OR “perioperative stress” OR “endocrine stress response” OR “immune activation” OR “metabolic adaptation”, combined with “surgery” OR “operative procedure” OR “perioperative period”, and further refined by terms related to hormonal, cytokine, or metabolic changes. Boolean operators (AND/OR), phrase matching, and truncation techniques were applied to increase sensitivity and specificity.

Manual searching supplemented the electronic searches. This included screening the reference lists of included studies and reviewing recent issues of high-impact journals such as Clinical Endocrinology, Acta Anaesthesiologica Scandinavica, Nature Reviews Endocrinology, and JAMA Surgery. Relevant abstracts and proceedings from major surgical and anesthesia conferences were also reviewed to ensure completeness. Two independent reviewers conducted all searches and screenings, and any disagreements were resolved through discussion or adjudication by a third reviewer.

Study selection

All retrieved titles and abstracts were screened for relevance to the endocrine, immune, and metabolic responses to surgical stress. Full-text articles were then reviewed to confirm eligibility based on predefined criteria. The PRISMA flow diagram outlines the number of records identified, screened, included, and excluded, along with the rationale for exclusion. This multi-stage selection process ensured that only studies meeting the methodological and thematic criteria were incorporated into the synthesis.

Data extraction and analysis

Data extraction was performed using a standardized template that captured study characteristics, participant demographics, surgical context, physiological outcomes, and major findings. For narrative and mechanistic reviews, emphasis was placed on extracting conceptual frameworks, mechanistic insights, and integrative interpretations that linked endocrine, immune, and metabolic pathways. Quantitative findings from empirical studies were synthesized descriptively where appropriate, while heterogeneous methodological designs and varied outcome measures were integrated using a narrative synthesis approach. This allowed the identification of recurring physiological patterns, mechanistic pathways, and clinically relevant implications in the context of surgical stress.

Quality assessment and certainty of evidence

The methodological rigor and risk of bias of the included studies were appraised using validated instruments. Observational and non-randomized studies were evaluated using the Risk of Bias in Non-randomized Studies of Interventions (ROBINS-I) tool [24], while RCTs were assessed with the Cochrane Risk of Bias (ROB) 2.0 framework [25]. Systematic reviews were appraised using the Assessment of Multiple Systematic Reviews 2 (AMSTAR 2) [26]. Narrative and mechanistic reviews were evaluated qualitatively based on clarity of purpose, comprehensiveness of the literature, mechanistic depth, and relevance to surgical stress physiology. Certainty of evidence across study designs was assessed using the GRADE (Grading of Recommendations, Assessment, Development, and Evaluations) framework [27], taking into account methodological limitations, consistency, directness, precision, and publication bias.

Risk of bias assessment

Risk of bias assessments contributed directly to the interpretation and weighting of evidence in the synthesis. Studies judged as low risk were given greater emphasis in shaping the mechanistic and clinical conclusions. Evidence from studies with moderate or unclear risk was interpreted cautiously and contextualized alongside higher-quality data. This approach ensured that the final synthesis accurately reflected the strength and reliability of the available evidence.

Results

Search Results

**Figure 1:**
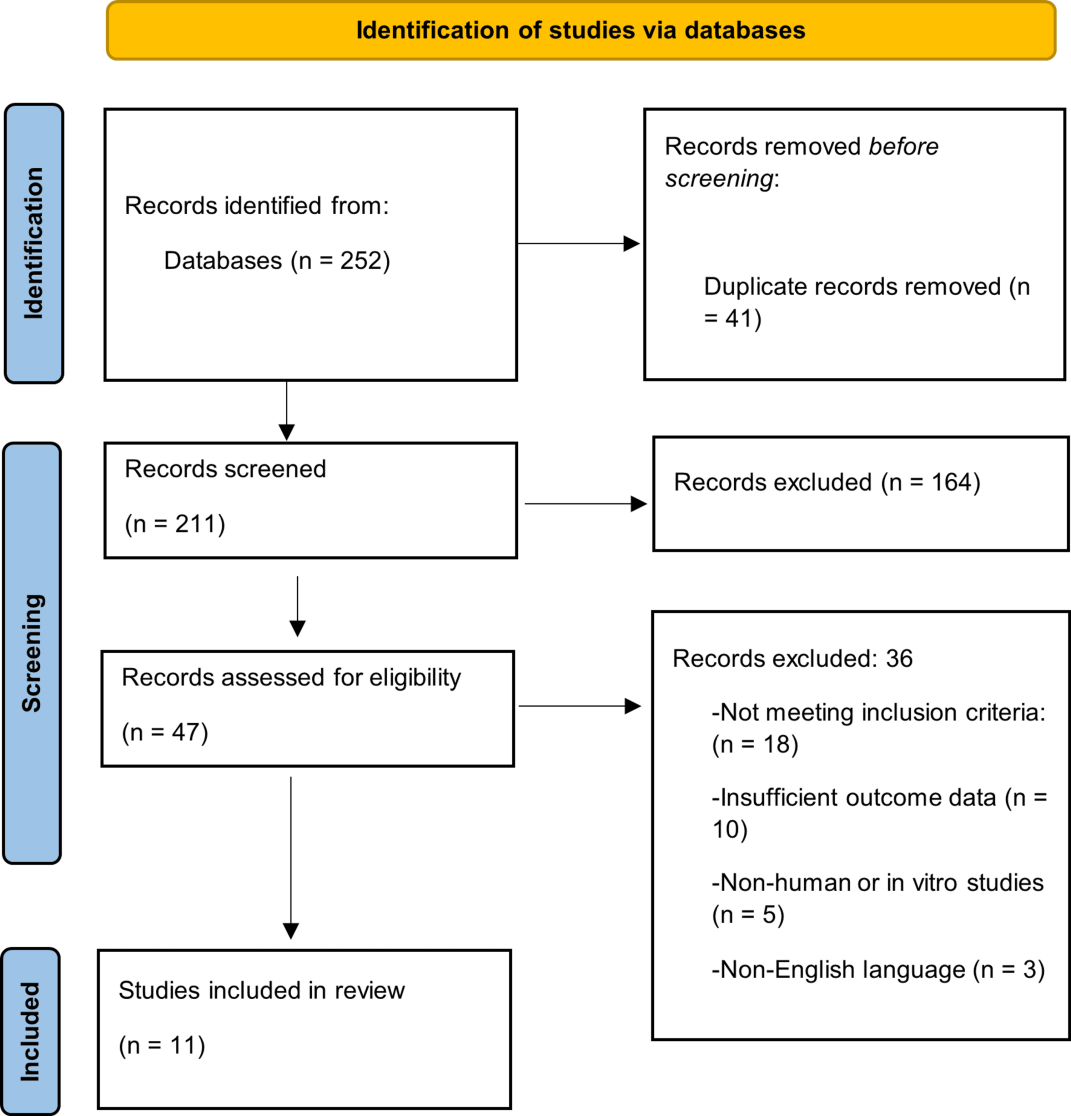
PRISMA flow diagram. PRISMA: Preferred Reporting Items for Systematic Reviews and Meta-Analyses.

The five main electronic databases used for the thorough literature search were Web of Science, PubMed, Scopus, ScienceDirect, and Google Scholar, together with manual screening of reference lists initially identified. Between January 2015 and March 2025, 252 records were released. There were 211 studies left for title and abstract screening after 41 duplicates were eliminated. Of these full-text articles, 47 were acquired for a comprehensive eligibility assessment. Following full-text review in compliance with the established inclusion and exclusion criteria, 11 studies were added to the final synthesis. Figure 1 shows the PRISMA flow diagram of study selection.

A wide variety of study designs, such as RCTs, were included in the included papers: prospective and retrospective cohort studies, systematic reviews, and narrative or mechanistic reviews provided integrative insights into the surgical stress response. Most investigations focused on major abdominal, cardiovascular, orthopedic, and oncologic surgical procedures, with primary outcome measures encompassing cortisol dynamics, cytokine profiles, glucose metabolism, immune modulation, and clinical recovery indices. These studies collectively offered a comprehensive perspective on the endocrine, immune, and metabolic alterations that characterize the physiological response to surgical stress. Table 1 summarizes the key findings and characteristics of the studies included in this review.

**Table 1:** Characteristics of the included studies. GI: gastrointestinal; ERAS: Enhanced Recovery After Surgery; HPA: hypothalamic-pituitary-adrenal; TNF-α: tumor necrosis factor-alpha; T2DM: type 2 diabetes mellitus.

Study (year)	Study design	Population/sample	Surgical context	Key outcomes measured	Major findings
Cusack & Buggy (2020) [1]	Narrative review	NA	General surgical procedures	HPA axis activation, catecholamine response	Demonstrated rapid activation of HPA and sympathetic systems; identified anesthetic techniques that modulate hormonal surge and improve outcomes.
Helander et al. (2019) [2]	Cohort study	240 adult patients	Abdominal & orthopedic surgeries	Metabolic rate, glucose metabolism, and insulin resistance	Perioperative metabolic reprogramming leads to insulin resistance in 60–80% of cases; early nutritional and glycemic interventions improve recovery.
Carli (2015) [3]	Randomized controlled trial	120 patients	ERAS vs. conventional care (colorectal surgery)	Insulin sensitivity, glucose levels, and length of stay	ERAS protocols significantly reduced insulin resistance and shortened hospital stay; metabolic optimization improves outcomes.
Prete et al. (2018) [5]	Systematic review & meta-analysis	32 studies, >1500 patients	Various major surgeries	Cortisol dynamics, endocrine stress markers	Cortisol peaks 4–24 hours postoperatively; the magnitude correlates with invasiveness and predicts complications such as hyperglycemia and delayed wound healing.
Ashley & Demas (2017) [6]	Mechanistic review	NA	Surgical stress physiology	Neuroendocrine–immune interactions	Detailed cross-talk between HPA axis, immune activation, and metabolic pathways; suggested integrated models for stress modulation.
Crosson (2018) [9]	Clinical implementation study	150 patients	ERAS implementation	Length of stay, complication rates, stress biomarkers	ERAS programs consistently attenuated physiological stress markers and improved postoperative outcomes across multiple surgery types.
González-Díaz et al. (2017) [11]	Clinical review	NA	Mixed surgical cohorts	Adaptive immunity markers, T-cell function	Documented perioperative immunosuppression and increased infection risk; highlighted psychoneuroimmunological modulation as a therapeutic target.
Scott et al. (2015) [12]	Prospective observational study	180 GI surgery patients	Gastrointestinal surgery	Cytokine kinetics (IL-6, TNF-α, IL-10), leukocyte activation	Identified cytokine surge within six hours post-incision; imbalance linked to increased risk of sepsis and delayed healing.
Hübner et al. (2016) [16]	Pilot study	85 GI surgery patients	Major gastrointestinal procedures	Albumin kinetics, inflammatory markers	Postoperative albumin drop strongly correlated with surgical stress and predicted complications and length of stay.
Sandoval & Patti (2023) [19]	Review & cohort synthesis	300 patients	Bariatric & metabolic surgery	Glucose utilization, hormonal adaptation	Bariatric surgery significantly alters glucose metabolism and stress hormone profiles; implications for T2DM remission.
Herman et al. (2016) [21]	Experimental & clinical review	NA	General surgical context	HPA regulation pathways, feedback mechanisms	Elucidated neuroendocrine regulation of stress response; highlighted potential therapeutic targets for modulating perioperative cortisol activity.

Endocrine response to surgical stress

Activation of the HPA Axis and Sympathetic System

The sympathetic-adrenal-medullary system and the HPA axis are quickly activated by surgical trauma, initiating the primary endocrine stress response. The normal cortisol levels increase during the period of 30-60 minutes after incision, peak between four to 24 hours, and slowly normalize within a few days. This surge promotes gluconeogenesis, sustains vascular tone, and regulates immune activity. Simultaneously, the release of catecholamines, particularly adrenaline and noradrenaline, increases in proportion to the extent and invasiveness of the surgical procedure, with longer or more complex operations eliciting a stronger response. This surge enhances cardiac output to maintain adequate tissue perfusion during surgical stress.

Hormonal Modulation and Clinical Correlates

Postoperative hyperglycemia, slow wound healing, and risk of infection are linked to persistent hypercortisolemia and excessive catecholamine activity, although in the short run, they are protective. Abnormal secretion of antidiuretic hormone (ADH) and growth hormone (GH) also helps in the retention of fluids, electrolyte imbalance, and metabolic disorders. The main endocrine alterations that can be seen during the perioperative period and the related clinical outcomes are summarized in Table 2.

**Table 2:** Summary of endocrine changes and clinical correlations. HPA: hypothalamic-pituitary-adrenal.

Hormone/pathway	Typical perioperative pattern	Physiological role	Clinical correlates and outcomes	Key references
Cortisol (HPA axis)	Rapid rise within 30–60 minutes post-incision, peaking at 4–24 hours; prolonged elevation in extensive surgery	Stimulates gluconeogenesis, mobilizes energy, and modulates immune response	Hyperglycemia, impaired wound healing, increased infection risk, and delayed recovery	[1,5]
Catecholamines (adrenaline, noradrenaline)	Surge within minutes of incision, sustained elevation during surgery, and gradual decline postoperatively	Increases cardiac output, vascular tone, and glucose mobilization	Tachycardia, hypertension, metabolic stress, and postoperative insulin resistance	[1,2]
Antidiuretic hormone (ADH)	Elevated during the intraoperative and early postoperative period	Enhances water retention, maintains blood pressure under hypovolemia	Fluid retention, electrolyte imbalance (hyponatremia), edema	[2,3]
Growth hormone (GH)	Transient elevation perioperatively, with variability based on surgical stress and anesthesia type	Promotes lipolysis, protein synthesis, and metabolic adaptation	Catabolic imbalance, postoperative nitrogen loss, and muscle wasting	[11]
Prolactin	Mild to moderate increase perioperatively due to stress activation	Modulates immune function and stress adaptation	Possible link to delayed immune recovery, though clinical impact remains under investigation	[11,21]

Immune system modulation

Innate Immune Activation

An important part of the innate immune activation is the rapid activation of the innate immune system, which is a major part of the surgical stress response. Most studies indicate that there is an initial surge of pro-inflammatory cytokines, including tumor necrosis factor-alpha (TNF-alpha), IL-1, and IL-6, within hours following surgical injury. This cytokine cascade promotes leukocyte recruitment, endothelial activation, and tissue repair, with the extent of release being closely related to the extent of tissue damage and blood loss.

Adaptive Immune Suppression

**Figure 2:**
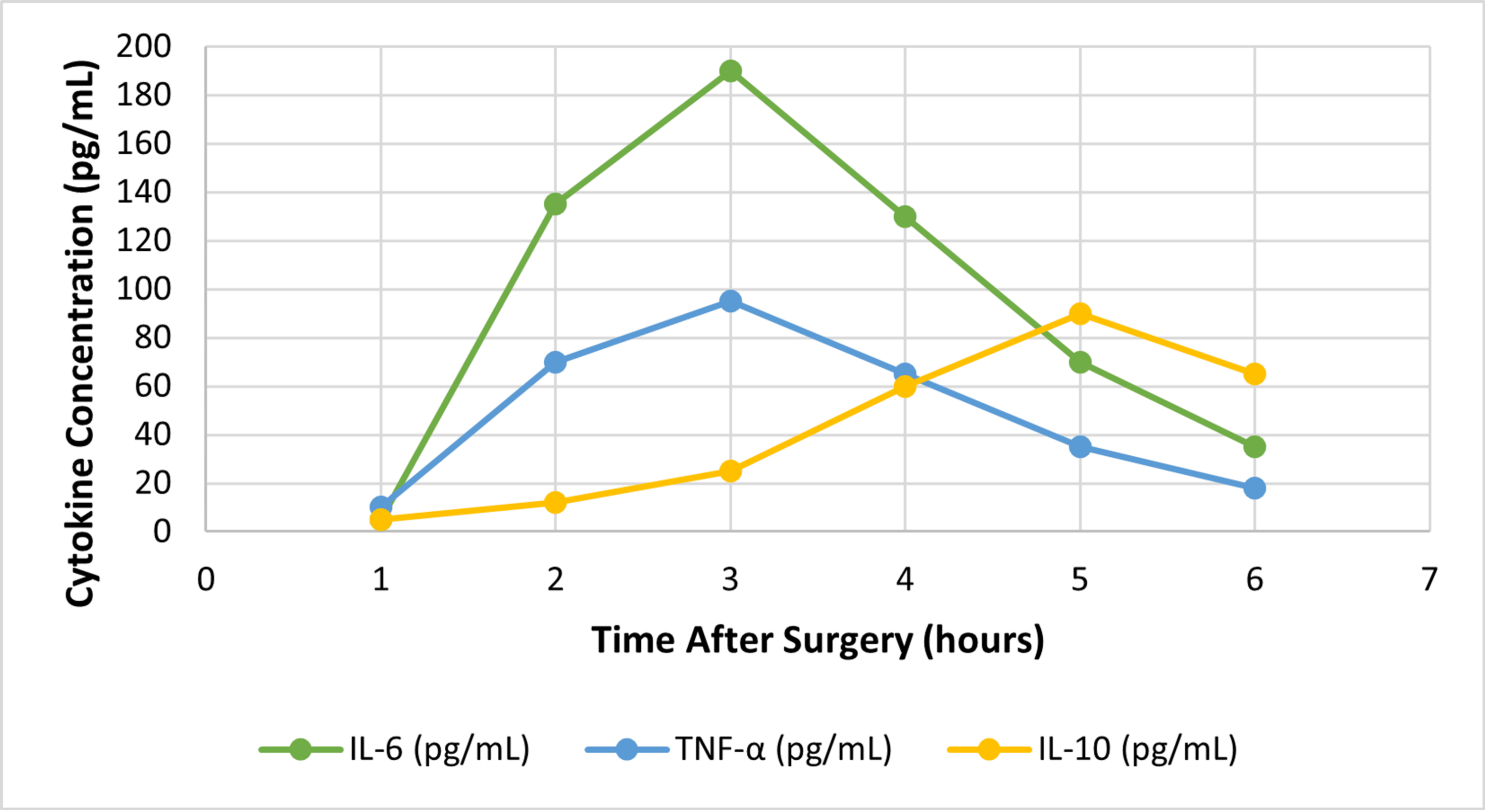
Cytokine dynamics post surgery. The figure was created by the authors. TNF-α: tumor necrosis factor-alpha; IL: interleukin.

At the same time, adaptive immunity will be transitorily suppressed, usually within the first 48-72 hours postoperatively. In addition, decreases in T-cell proliferation, lymphocyte counts, and antigen presentation capacity, together with increases in the concentration of inflammation-suppressing cytokines, such as IL-10, contribute to an increased risk of infection and delayed wound healing. Figure 2 demonstrates the dynamics of cytokines after surgery, where there is an example of the time course for levels of TNF-alpha, IL-10, and IL-6 after surgical treatment.

Metabolic adaptations and dysregulation

Energy Mobilization and Substrate Utilization

The surgical stress response causes major metabolic reprogramming in response to the increased energy demands. Gluconeogenesis and glycogenolysis provide glucose to the vital organs, and lipolysis and proteolysis provide fatty acids and amino acids as other substrates for energy. Although these processes are initially adaptive, they may result in negative nitrogen balance, catabolic muscle breakdown, and weight loss during long recovery.

Insulin Resistance and Glycemic Control

Perioperative insulin resistance, present in up to 80% of surgical patients, is usually maximal within 24-48 hours following surgery, is associated with delayed wound healing and hyperglycemia, and is associated with increased risk of infection. Metabolic outcomes have been significantly improved by such measures as nutritional optimization, preoperative carbohydrate loading, and perioperative glucose control. Table 3 summarizes the main metabolic changes that occur during the perioperative period, their physiologic significance, related clinical consequences, and possible intervention strategies.

**Table 3:** Metabolic changes and clinical implications. ↑: increase/elevated; ↓: decrease/reduced; GH: growth hormone; ERAS: Enhanced Recovery After Surgery.

Metabolic pathway/process	Perioperative change	Physiological role	Clinical consequences	Intervention strategies	Key references
Glucose metabolism	↑ Gluconeogenesis and glycogenolysis due to elevated cortisol and catecholamines	Ensures continuous glucose supply for vital organs during stress	Hyperglycemia, impaired wound healing, increased infection risk	Perioperative glucose monitoring, insulin therapy, and ERAS-based carbohydrate loading	[2,5]
Insulin sensitivity	↓ Insulin sensitivity (insulin resistance) peaking 24–48 hours post surgery	Shifts glucose toward essential tissues (brain, immune cells)	Persistent hyperglycemia, delayed recovery, metabolic imbalance	Early mobilization, optimized anesthesia, and preoperative carbohydrate loading	[19]
Protein metabolism	↑ Proteolysis and nitrogen loss to provide amino acids for gluconeogenesis and tissue repair	Supports energy production and wound healing	Muscle wasting, delayed functional recovery, prolonged convalescence	Perioperative nutrition, amino acid supplementation, and early enteral feeding	[18]
Lipid metabolism	↑ Lipolysis and free fatty acid mobilization driven by catecholamines	Provides alternative energy substrates under stress	Lipotoxicity, insulin resistance exacerbation, metabolic inflammation	Nutritional modulation, anti-inflammatory strategies, lipid monitoring	[15,17]
Energy expenditure	↑ Resting metabolic rate and catabolic activity	Meets increased energy demand for immune response and tissue repair	Negative energy balance, fatigue, delayed recovery	Caloric optimization, early nutritional support, multimodal ERAS interventions	[3]
Hormonal regulation	↑ Cortisol, GH, catecholamines; altered leptin and adiponectin signaling	Coordinates energy redistribution and substrate utilization	Endocrine-metabolic dysregulation, prolonged catabolism	Hormonal modulation, stress attenuation techniques	[5,28]

Neuroendocrine-immune-metabolic interactions

One of the major conclusions of this review is the widespread crosstalk between the immunological, metabolic, and neuroendocrine systems during the perioperative period. Cortisol and catecholamines have immunosuppressive effects by downregulating pro-inflammatory signaling and cytokines (e.g., IL-6) modulate endocrine activity and metabolic substrate utilization. Metabolic signals, such as glucose and free fatty acids, in turn, affect immune cell functioning and hormonal feedback processes.

The networks of these interconnections indicate that the surgical stress response is a system-level phenomenon, which has adaptive feedback loops that promote homeostasis in response to stress but can be the source of pathophysiological complications when deregulated.

Clinical correlates and outcome associations

Several biomarkers, including peak cortisol, IL-6 concentrations, perioperative glucose levels, postoperative albumin decline, C-reactive protein (CRP), and lymphocyte count, emerged as strong predictors of adverse postoperative outcomes such as infection, delayed wound healing, and prolonged hospitalization. These biomarkers reflect the integrated activity of neuroendocrine, immune, and metabolic pathways during surgical stress and serve as valuable indicators for early risk stratification and targeted intervention. Table 4 summarizes the predictive biomarkers, their perioperative thresholds, associated outcomes, and clinical implications.

**Table 4:** Predictive biomarkers and clinical outcomes. ERAS: Enhanced Recovery After Surgery; IL: interleukin; HPA: hypothalamic-pituitary-adrenal; SIRS: systemic inflammatory response syndrome.

Biomarker	Typical perioperative threshold	Clinical significance	Associated outcomes	Intervention implications	References
Cortisol (serum)	Peak: >500–600 nmol/L within 4–24 hours post surgery	Reflects HPA axis activation and surgical stress intensity	Prolonged hyperglycemia, delayed wound healing, increased infection risk	Early detection of patients at risk for immunological and metabolic issues is facilitated by tracking cortisol trends; the potential for glucocorticoid modulation	[1]
IL-6 (serum/plasma)	Peak: >80–100 pg/mL within 12–24 hours postoperatively	Indicates pro-inflammatory response magnitude	Higher incidence of SIRS, sepsis, and prolonged hospitalization	Early cytokine profiling helps guide immunomodulatory interventions and identify the risk of systemic inflammatory complications	[11,12]
Perioperative glucose	>180 mg/dL in the first 24–48 hours post surgery	A marker of insulin resistance and metabolic stress	Poor wound healing, increased infection rates, and longer ICU stay	Tight glycemic control (insulin infusion protocols, ERAS carbohydrate loading) improves outcomes	[5]
Postoperative albumin drop	>10–15% decrease from baseline within 24–48 hours	Reflects systemic inflammation and metabolic response	Increased length of stay, postoperative complications, and higher morbidity risk	Albumin monitoring serves as a surrogate marker for surgical stress severity and predicts adverse clinical trajectories	[16]
C-reactive protein (CRP)	>150 mg/L at 48–72 hours post surgery	Nonspecific marker of inflammatory burden	Delayed recovery, infection, prolonged hospital course	Routine CRP surveillance supports postoperative risk stratification	[13,14]
Lymphocyte count	<1.0 × 10⁹/L within 48 hours	Indicates adaptive immune suppression	Increased nosocomial infection risk, slower wound repair	Immune monitoring may guide early immunonutrition or adjuvant therapy	[6,11]

Quality assessment and risk of bias

The included studies had moderate to high methodological quality. Due to the nature of surgical procedures, some RCTs showed unclear risk in blinding participants and staff, but the majority demonstrated minimal bias risk in the domains of allocation concealment, random sequence creation, and reporting of primary outcomes. According to the ROBINS-I tool, observational studies were assessed as having a modest risk of bias, mainly owing to potential confounding factors, variability in patient populations, and incomplete adjustment for baseline characteristics. Despite these limitations, the overall evidence base was considered robust and reliable, with a consistent direction of effect across studies. According to the GRADE framework, the degree of evidence certainty varied from moderate to high for endocrine and metabolic outcomes and from low to moderate for immune-related findings, reflecting heterogeneity in outcome measurement and follow-up duration. Of the 11 included studies, five empirical studies (RCTs, cohorts, or pilot studies) were eligible for formal risk-of-bias evaluation, while the remaining six narrative or mechanistic reviews were appraised qualitatively. Based on the ROB 2.0 and ROBINS-I criteria, the risk of bias evaluation and quality assessment of the included research are displayed in Table 5.

**Table 5:** Risk of bias assessment. RCT: randomized controlled trial.

Study	Design	Randomization	Allocation concealment	Blinding	Outcome reporting	Confounding	Overall risk
Prete et al. (2018) [5]	Meta-analysis	N/A	N/A	N/A	Low	N/A	Low
Helander et al. (2019) [2]	Cohort	N/A	N/A	N/A	Low	Moderate	Moderate
Carli (2015) [3]	RCT	Low	Low	Unclear	Low	N/A	Low
Hübner et al. (2016) [16]	Pilot study	N/A	N/A	N/A	Moderate	Moderate	Moderate
González-Díaz et al. (2017) [11]	Review	N/A	N/A	N/A	Low	N/A	Low

Discussion

The surgical stress response is a multifaceted, tightly coordinated physiological phenomenon involving neuroendocrine, immune, and metabolic systems. These adaptive mechanisms preserve homeostasis and support tissue repair, yet excessive or prolonged activation predisposes patients to adverse outcomes. A central feature of this response is the rapid activation of the HPA axis and the sympathetic-adrenal-medullary pathways following tissue injury [1]. The resulting surge in cortisol and catecholamines mobilizes metabolic reserves, modulates vascular tone, and influences immune dynamics, enabling acute adaptation. However, sustained hypercortisolemia and excessive catecholamine activity disrupt glucose regulation and immune efficiency, contributing to postoperative hyperglycemia, impaired wound healing, and heightened vulnerability to infection [5].

The immune response exhibits an equally complex temporal pattern. Surgical trauma initiates an early pro-inflammatory cascade characterized by elevations in TNF-α, IL-1β, and IL-6, which promote leukocyte recruitment, endothelial activation, and tissue repair [12]. This is followed by a compensatory anti-inflammatory phase marked by lymphopenia, reduced T-cell proliferation, and increased IL-10 secretion, which helps limit tissue damage but increases susceptibility to nosocomial infections and delays wound healing. These dynamic shifts underscore the importance of optimizing the timing and intensity of immunomodulatory interventions to maintain an appropriate balance between pro- and anti-inflammatory activity rather than applying indiscriminate suppression.

Metabolic reprogramming represents another essential element of the surgical stress response. Increased gluconeogenesis and glycogenolysis ensure continuous glucose availability to vital organs, while enhanced lipolysis and proteolysis provide metabolic substrates for tissue repair. Although these adaptations sustain short-term metabolic demands, they contribute to negative nitrogen balance, muscle catabolism, and prolonged recovery periods. The development of perioperative insulin resistance further disrupts metabolic homeostasis and exacerbates hyperglycemia-related complications. Evidence indicates that perioperative nutritional optimization, glycemic control, preoperative carbohydrate loading, and early enteral feeding can mitigate these metabolic derangements and improve outcomes.

Beyond these established pathways, the broader physiological context also influences the stress response. Neuroendocrine activation and inflammatory fluctuations interact with the gut microbiota, which undergoes significant shifts following surgical manipulation and anesthesia [29]. Crosstalk between cytokines, neurotransmitters, and hormonal mediators further shapes recovery trajectories, reflecting a multidirectional network that integrates neural, endocrine, and immune signals [30]. Pre-existing metabolic vulnerability may magnify these effects, as chronic stress is known to impair metabolic flexibility and increase the risk of insulin resistance and related disorders [31]. Additionally, host stress mediators reshape the cutaneous microbiome, influencing wound healing quality and infection risk [32], while microbial endocrinology principles demonstrate that endogenous catecholamines can modulate microbial proliferation and virulence, linking neuroendocrine stress signals to postoperative infectious complications [33].

Lipid mediators such as eicosanoids also play a role in postoperative physiology, contributing to inflammatory signaling and tissue remodeling; dysregulation of these pathways may further amplify surgical inflammation [34]. Individual variability in innate and adaptive immune responses, influenced by genetic, behavioral, and environmental factors, modifies the magnitude of surgical inflammation and recovery profiles [35]. Psychological and sensory factors additionally shape perioperative stress reactivity, influencing neuroendocrine output and inflammatory tone [36]. Interventions such as perioperative music therapy have demonstrated measurable reductions in anxiety, sympathetic activation, and inflammatory markers, underscoring the multimodal nature of stress modulation [37].

Cognitive outcomes are increasingly recognized as an important dimension of the postoperative stress response. Cytokine surges, neuroinflammation, and anesthetic interactions contribute to perioperative neurocognitive disorders, with recent evidence emphasizing cytokine-driven neural alterations as a major mechanism [38]. Metabolic stress and prolonged inflammatory signaling may induce microglial priming and “inflammatory memory,” which can perpetuate cognitive dysfunction [39]. Emerging pharmacological insights also highlight natural compounds such as terpinen-4-ol, which demonstrate anti-inflammatory and immunomodulatory potential relevant to perioperative physiology [40], while bioactive molecules capable of altering microbial quorum-sensing pathways may indirectly regulate inflammation and influence postoperative wound recovery and host-microbe interactions [41].

The clinical translation of these mechanistic insights is well reflected in Enhanced Recovery After Surgery (ERAS) programs. ERAS-based interventions, which combine preoperative optimization with minimally invasive surgical techniques, multimodal analgesia, and early mobilization, have consistently demonstrated reductions in endocrine activation, modulation of inflammatory pathways, and improvement in metabolic outcomes [3]. These physiological improvements align with reductions in postoperative complications, shortened hospital stays, and faster functional recovery, illustrating the value of a systems-based approach to perioperative care [9]. Despite notable progress, heterogeneity in study design, outcome measures, and mechanistic frameworks limits the generalizability of current findings. Future research should prioritize integrative, multi-omic methods capable of linking molecular pathways to clinical phenotypes while accounting for patient-specific factors such as age, comorbidities, and genetic predisposition.

The surgical stress response is thus best understood as a complex, interconnected set of adaptive processes that are essential for survival but potentially harmful when excessive or prolonged. A deeper understanding of the integrated neuroendocrine, immune, and metabolic dynamics provides a foundation for targeted, patient-centered perioperative strategies. Through biomarker-informed interventions, metabolic and immune modulation, and comprehensive ERAS-driven optimization, perioperative medicine can advance toward precision-based approaches that mitigate surgical risk while enhancing recovery and long-term outcomes.

Limitations and future recommendations

Although this review forms a good source of information, it should be noted that there are a number of limitations to the review. The included studies' significant differences in design, patient demographics, surgical techniques, and outcome measures restrict the findings' capacity to be compared and synthesized. The lack of consistency in the cytokine measurements, hormone tests, and the methodology of the metabolic assessment also adds to the interpretation of data. Besides, the majority of the evidence available is limited to the short-term perioperative studies, which offer minimal insights into the effect of these physiological responses on the long-term clinical outcome. The scope of evidence used could also have been limited by potential publication bias and the non-English studies.

Standardized methods of measuring endocrine, immune, and metabolic responses should be a priority in future studies to enhance reproducibility and commonality between studies. Mechanistic understanding of the surgical stress response needs to be complemented with integrative, longitudinal studies that indicate how the response relates to clinical outcomes. Also, predictive models that include biomarkers and patient-specific risk factors, as well as the use of procedural variables, and specific immunometabolic and nutritional interventions, are significantly promising to expand individualized perioperative care and patient outcomes.

## Conclusions

The review is a novel addition to the literature because it is integrative and provides a holistic perception of all physiological processes of the surgical stress response, and it combines endocrine, immune, and metabolic perspectives under a single conceptual roof. Unlike previous studies that have tended to study these pathways separately, this study offers a description of how these pathways interact, control one another, and determine the result of the perioperative period. It broadens the corpus of information already available on the role of these systems in the recovery process, correlating with hormonal maintenance, immune regulation, and metabolic adaptation. Another important aspect of the review is that it demonstrates the potential use of relevant clinical biomarkers and predictive indicators in risk stratification and personalized intervention. In addition to that, it helps to uncover the significant voids in the current literature, encouraging future studies that ought to be dedicated to the correlation of molecular processes with clinical practice to facilitate accurate perioperative treatment. By adopting this multidimensional method, the review not only enhances scientific understanding but also creates a solid base for the creation of specific strategies to reduce surgical stress and improve patient outcomes.
